# Case Report: The widening genetic and phenotypic spectrum of ultra-rare PDE4D-related acroscyphodysplasia

**DOI:** 10.3389/fmed.2025.1623593

**Published:** 2025-08-22

**Authors:** Anna Morgul, Margarita Sharova, Vladimir Kenis, Maria Orlova, Oxana Ryzhkova, Tatiana Markova

**Affiliations:** ^1^Research Centre for Medical Genetics, Moscow, Russia; ^2^H. Turner National Medical Research Center for Children’s Orthopedics and Trauma Surgery of the Ministry of Health of the Russian Federation, Saint Petersburg, Russia

**Keywords:** acroscyphodysplasia, *PDE4D*, scaphoid knee deformities, brachydactyly, growth retardation

## Abstract

Acroscyphodysplasia (ASD) is an ultra-rare skeletal dysplasia characterized by severe brachydactyly, metaphyseal scaphoid knee deformities, growth retardation, and intellectual disability. To date, only seven cases of ASD have been reported, all associated with missense variants in the *PDE4D* gene. We report a 7-year-old girl with ASD features, including midface hypoplasia, severe growth retardation (−4.81 Shwachman–Diamond syndrome (SDS) height), progressive postnatal development of “cup-shaped” knee metaphyses, and unilateral humeral bowing, demonstrating mosaic growth plate involvement. Whole-genome sequencing revealed a novel *PDE4D* missense variant (c.934C>T, p. Leu312Phe) in the upstream conserved region 2 (UCR2) autoinhibitory domain, which is distinct from known acrodysostosis-associated variants. Expanding the clinical and radiological characteristics, as well as the mutation spectrum of *PDE4D*-related ASD, is crucial for understanding syndrome variability, aiding in earlier detection, and improving recurrence risk assessment.

## Introduction

Acroscyphodysplasia (ASD; Online Mendelian Inheritance in Man (OMIM) catalog number: 250215) is an ultra-rare autosomal dominant form of skeletal dysplasia characterized by metaphyseal scaphoid deformity of the knee joints, severe brachydactyly with cone-shaped epiphyses, distinct growth retardation, facial dysostosis with nasal hypoplasia, and intellectual disability (1, 2). The term metaphyseal ASD was introduced by Verloes et al. (3). The authors consolidated clinical data from four patients, two of whom had been previously reported by Bellini et al. and Jequier et al. They identified distinct radiological features, including a characteristic morphology of the knee joints characterized by cup-shaped metaphyses with embedded epiphyses (termed “skypho,” derived from the Greek word for “cup”) and micromelia of the lower limbs. The prefix “acro-” denotes the involvement of the distal parts of the limbs (3–5). ASD has since been reported as a phenotypic feature of acrodysostosis type 2 (associated with *PRKAR1A* and *PDE4D* genes) and pseudohypoparathyroidism (associated with *GNAS* gene) (6).

The etiopathogenesis of ASD is not yet fully elucidated. To date, only seven cases of ASD have been reported, all associated with missense variants in the *PDE4D* gene, which is also implicated in the etiology of acrodysostosis type 2, with or without hormonal resistance (OMIM *#* 614613) (1, 2, 6, 7). Previous reports have suggested that the *PDE4D* gene may be responsible for ASD, representing a more severe form of skeletal dysplasia encompassing acrodysostosis. The protein product of the *PDE4D* gene is cAMP-specific phosphodiesterase 4D (*PDE4D*), a key component of the GPCR-cAMP-PKA signaling pathway, which mediates cellular hormonal control and regulates hypertrophic differentiation of growth plate chondrocytes (8, 9). Given the limited number of reported *PDE4D*-related ASD cases, accumulating data on new ASD cases will contribute to a deeper understanding of its clinical manifestations, pathogenic mechanisms, and optimization of diagnostic approaches.

In this study, we analyzed the clinical and radiological characteristics of ASD in a 7-year-old girl carrying a novel *de novo* heterozygous variant in the *PDE4D* gene.

## Materials and methods

A comprehensive examination was conducted on a 7-year-old female proband presenting with phenotypic features of skeletal dysplasia, developmental delay, and facial dysmorphisms. To refine the diagnosis, a comprehensive clinical-genealogical analysis was performed, along with X-rays of the spine, hip joints, and long bones of the extremities, as well as molecular genetic testing.

Written informed consent was obtained from the proband’s parents. The study was performed in accordance with the Declaration of Helsinki and approved by the Institutional Review Board of the Research Centre for Medical Genetics, Moscow, Russia.

Blood samples were collected from the proband and his unaffected parents, and genomic DNA was extracted by standard methods using a Wizard Genomic DNA Purification Kit (Promega, WI, United States).

Whole-genome sequencing was performed using a DNBSEQ-G400 instrument in paired-end mode (2 × 150 bp), with an average on-target coverage of 30×. Library preparation was carried out using the MGIEasy FS PCR-Free DNA Library Prep Set (BGI, Beijing, China). Bioinformatic analysis was performed using an in-house software pipeline, as previously described [PubMed Identifier (PMID): 29504900], with modifications. In brief, quality control of raw reads was conducted using FastQC (version 0.11.5), followed by read mapping to the hg19 human genome assembly using minimap2 (version 2.24-r1122). Alignments were sorted, and duplicates were marked using the Picard Toolkit (version 2.18.14). Base recalibration and variant calling were performed using GATK3.8. Variant annotation was performed using the ANNOVAR tool (version 2018-04-16). Copy number variation (CNV) and structural variation (SV) analyses were performed using Manta (version 1.6.0). Further filtering was based on functional consequences and population frequencies, according to the American College of Medical Genetics and Genomics (ACMG) recommendations (10), as well as clinical relevance determined using the Human Phenotype Ontology database. The complementary DNA (cDNA) and protein positions in the *PDE4D* gene corresponded to transcript NM_001104631.2. Identified *PDE4D* variants were validated by Sanger sequencing according to standard protocols, using a 3500xL Genetic Analyzer (Applied Biosystems, Thermo Fisher Scientific, MA, United States).

## Clinical case

The proband was a 7-year-old girl who underwent clinical evaluation due to hyperactivity, delayed motor and speech development, short stature, obesity, and difficulties with independent ambulation. The parents were healthy, non-consanguineous, and had three older healthy daughters and one older healthy son. Prenatal ultrasound during the final weeks of gestation revealed a 2-week intrauterine growth retardation. The child was delivered by cesarean section at 40–41 weeks of gestation with a birth weight of 2,600 g (−2.09 SDS) and a length of 48 cm (−1.07 SDS). Craniofacial dysmorphisms, including hypertelorism and a saddle nose, were noted at birth. Brachydactyly of both hands and feet became obvious by 4 months of age. Due to the facial phenotype and brachydactyly, achondroplasia was suspected, and a screen for variants in the *FGFR3* gene was performed, yielding no positive outcome. Standard cytogenetic testing demonstrated a normal female karyotype 46, XX.

**Figure 1:**
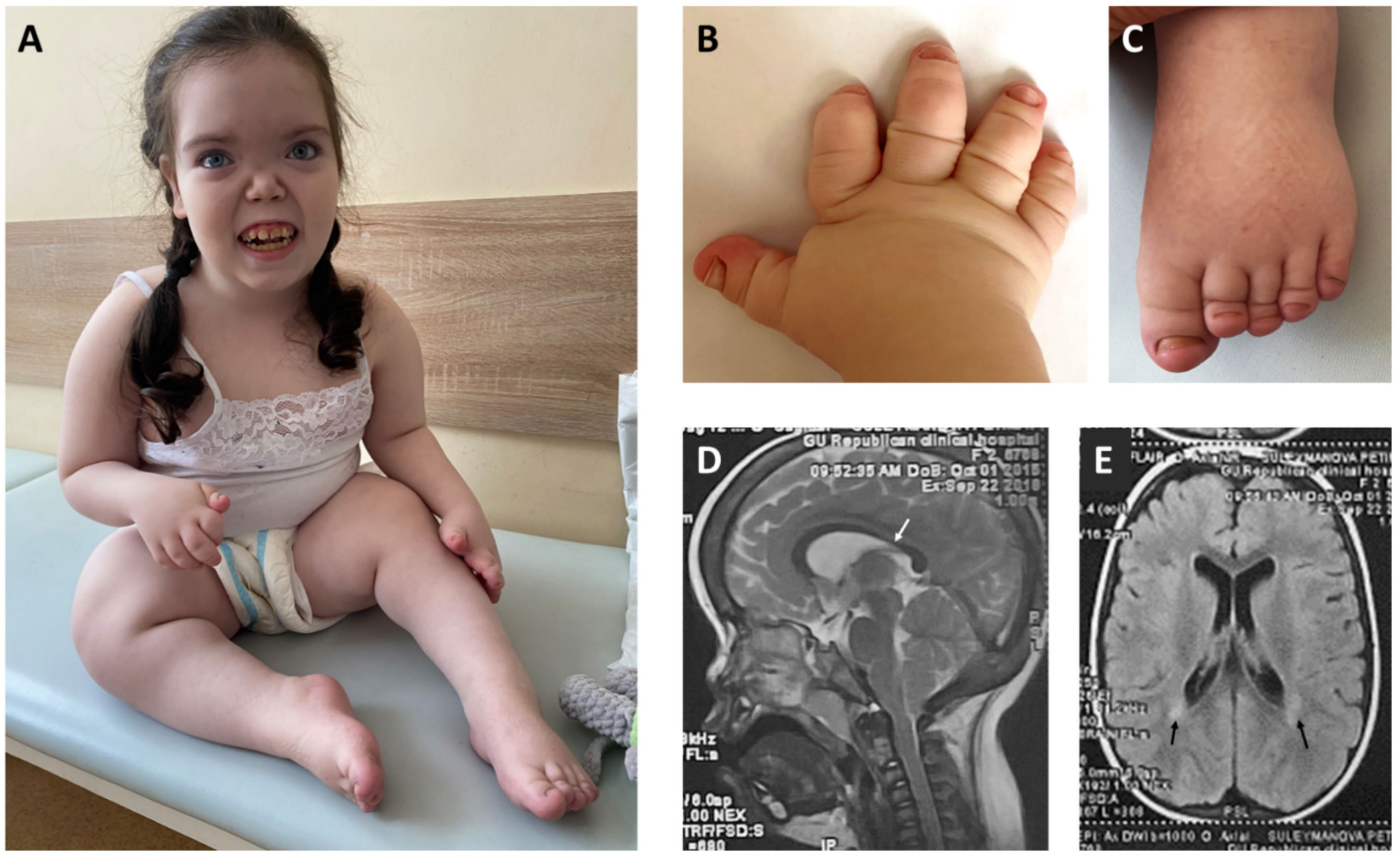
**(A)** - Facial features: broad forehead with prominent frontal bossing, hypertelorism, low nasal bridge, midface hypoplasia, long smooth philtrum, tented upper lip, protruding upper incisors, enamel hypoplasia, open mouth. Obesity, growth retardation with micromelia, knee joint contractures. **(B,C)** – Severe brachydactyly with nail hypoplasia. **(D)** – Midsagittal view of brain MRI – thinning of the isthmus of corpus callosum (white arrow). **(E)** – Axial view of brain MRI – periventricular leukomalacia adjacent to the external angles of the lateral ventricles (black arrows).

The patient exhibited global developmental delay, with the following milestones: head control at 9 months, independent sitting at 18 months, and ambulation at 20 months of age. Expressive language was limited to vocalizations without meaningful speech. Receptive language skills were partially impaired (inconsistent responses to names and commands). Stereotypic hand-flapping movements were observed during periods of excitation. The patient remained dependent on spoon-feeding and lacked toilet training skills. Recurrent upper respiratory infections were documented in early childhood. During 1–6 years of age, the patient experienced febrile seizures (2–3 annual episodes). Video-EEG monitoring showed no epileptiform activity. Progressive weight gain led to a class-IV obesity diagnosis by 6 years of age. Progressive weight gain became clinically evident after 4 years of age, culminating in a diagnosis of stage-IV obesity by 6 years of age. Orthopedic evaluation revealed radiographic features suggestive of Cerebral, Ocular, Dental, Auricular, Skeletal (CODAS) syndrome, including bifid distal femoral morphology and nasal hypoplasia. Ophthalmological examination demonstrated retinal angiopathy. Brain magnetic resonance imaging (MRI) findings included corpus callosum hypoplasia, periventricular leukopathy (localized to the posterior horns of the lateral ventricles), Arnold–Chiari malformation type I, and hyperostosis of the calvarial bones (Figure 1C). Cardiac evaluation identified a 2.5-mm foramen ovale. Abdominal ultrasonography confirmed left renal lumbar ectopia.

Clinical evaluation at 7 years of age revealed severe growth retardation (height 95 cm, −4.79 SDS), excessive body weight (30 kg, body mass index (BMI) + 4.17 SDS), and a trend toward macrocephaly (head circumference 54 cm, +1.93 SDS). The patient exhibited facial features such as flat face, severe midface hypoplasia, flat nasal bridge, hypertelorism, telecanthus, and disproportionate skeletal features, such as rhizomelic limb shortening and brachydactyly of hands and feet, with marked shortening of distal phalanges and nail hypoplasia (Figures 1A–C). Neurological examination demonstrated flexion contractures of the knee joints, generalized muscle hypotonia, and an abnormal waddling gait characterized by knee flexion posture and forward trunk inclination. Brain MRI findings included thinning of the isthmus of the corpus callosum and periventricular leukomalacia (Figures 1D,E).

**Figure 2:**
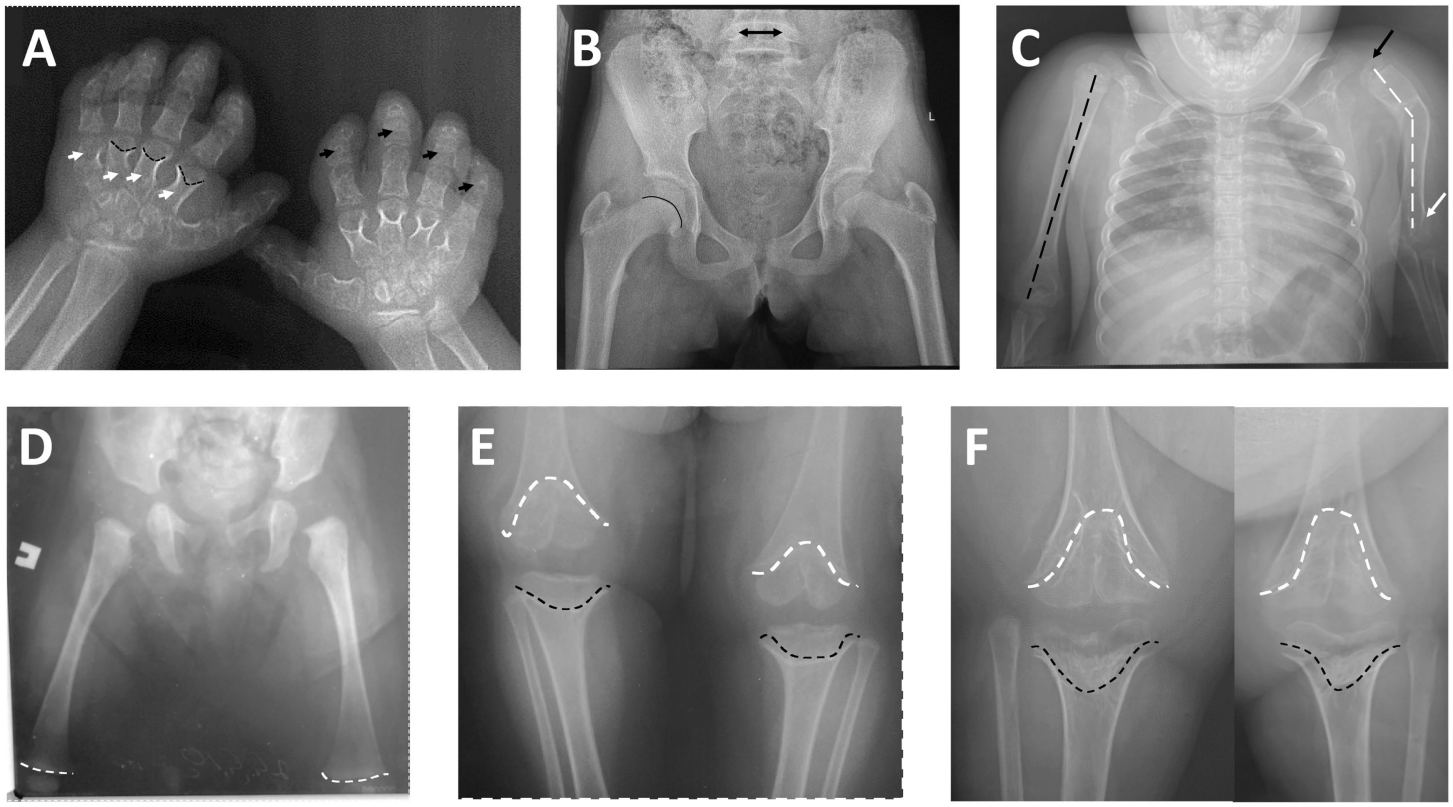
**(A)** Posteroanterior radiograph of the hands: short metacarpals (white arrows) and phalanges—mostly middle phalanges (black arrows), cone-shaped epiphyses of metacarpal bones (black broken lines). **(B)** Anteroposterior radiograph of the hips (at 7 years of age)—normal radiographic parameters, intact growth plate (black line) and normal interpedicular distance of L5 (black arrow). **(C)** Anteroposterior radiograph of the chest and upper limbs: normal axis of the right humerus (black broken line) with intact shoulder and elbow; severe axial deformity of the left humerus (black broken lines), subluxation of the left shoulder (black arrow), and deformity of the left elbow (white arrow). **(D)** Anteroposterior radiograph of the hips and femoral bones (age 3 months)—notice normal distal femoral growth plates (white broken lines). **(E)** Anteroposterior radiograph of the knee joints (at 3 years of age): remarkable epi-metaphyseal deformity (cone-shaped) of both distal femur (white broken lines) and proximal tibia (black broken lines). **(F)** Anteroposterior radiograph of the knee joints (at 7 years of age): progress of the epi-metaphyseal deformity (wedge-shaped) of both distal femur (white broken lines) and proximal tibia (black broken lines).

The most striking radiological findings were demonstrated in the knees and hands. Interestingly, typical epi-metaphyseal deformity was not presented in the early radiographs—distal femoral epiphyseal growth plates were not deformed, and the shape of epiphyses was not changed at 3 months of age. At 3 years of age, anteroposterior radiographs of the knees already documented noticeable cone-shaped epiphyses of both distal femora and proximal tibiae, which progressed to the cupped metaphyseal deformity (typical scypho-deformity of the knees) at 7 years of age. Radiographs of the hips demonstrated normal radiographic parameters and intact growth plates. Spine radiographs show mild scoliosis and thoracolumbar kyphosis; interpedicular distance at L3–L5 remains unchanged. Severe bowing of the left humerus and valgus deformity of the elbow were found on the left side, with a relatively normal right upper limb. Short metacarpals and phalanges (mostly middle phalanges) and cone-shaped epiphyses of metacarpal bones were found on the radiographs of the hands (Figure 2).

Biochemical parameters of mineral metabolism, including total serum calcium, inorganic serum phosphorus, parathyroid hormone, and alkaline phosphatase levels, were found to be within the established reference ranges. However, a deficiency of vitamin D-25-OH was identified, with a concentration of 24.96 ng/mL, which falls below the normal range of 30–100 ng/mL.

Whole-genome sequencing identified a novel pathogenic variant in exon 7 of the *PDE4D* gene (c.934C>T), resulting in a heterozygous missense substitution, p. Leu312Phe. The identified variant was confirmed by Sanger sequencing in the proband’s DNA but was not present in the parents’ DNA, confirming it as *de novo*. This variant has been classified as likely pathogenic based on the ACMG guidelines (PM2, PM5, and PM1) (10).

## Discussion

**Figure 3:**
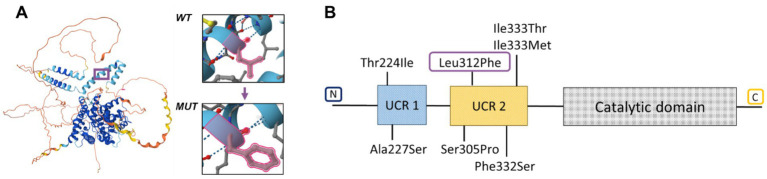
**(A)** Structure of protein PDE4D with amino acid substitution. **(B)** Genotype of patients with acroscyphodysplasia in PDE4D.

ASD is an ultra-rare skeletal dysplasia characterized by distinctive radiological findings such as cup-shaped metaphyses with embedded epiphyses (particularly evident in the knees), combined with characteristic clinical features including acrodysostosis-like brachydactyly and nasal hypoplasia, which together constitute the primary diagnostic criteria for this condition (2). Furthermore, short stature and intellectual disability have been consistently documented in all reported cases. In the presented case, a 7-year-old girl exhibited pronounced phenotypic and radiological features—including midface and nasal hypoplasia, metaphyseal cupping, brachydactyly, severe short stature (−4.81 SDS), and obesity—suggestive of a rare skeletal dysplasia with intellectual disability. Given the overlapping clinical manifestations with other hereditary disorders, the diagnosis of ASD was challenging due to its rarity. Thus, the presence of cognitive impairment combined with coarse facial features, obesity, short stature, and brachydactyly initially raised suspicion for CHOPS syndrome (OMIM catalog number: 616368). However, the proband lacked commonly associated cardiac and pulmonary anomalies, and radiographic skeletal findings were inconsistent with this diagnosis (11). In addition, the metaphyseal changes observed in the proband’s knee joints showed partial similarity to the characteristic ‘bifid’ appearance of the distal femur noted in CODAS syndrome (OMIM: 600373), which similarly presents with developmental delay, short stature, midface and nasal hypoplasia, and other congenital anomalies (12). Ultimately, whole-genome sequencing led to a definitive diagnosis by identifying a novel *de novo* heterozygous missense variant in exon 7 of the *PDE4D* gene (c.934C>T, p.Leu312Phe) that had not been previously reported. Most known pathogenic variants in this gene, which primarily cause acrodysostosis type 2, are missense mutations distributed throughout the coding sequence. However, previously described missense variants in ASD cases are located in the N-conserved domains UCR1 and upstream conserved region 2 (UCR2) (Figure 3B). These domains interact with the catalytic domain of the adjacent monomer to form an autoinhibitory domain that partially occludes the catalytic site (13, 15). The novel variant we detected results in the substitution of leucine (with its non-polar alkyl R-group) by phenylalanine (containing an aromatic R-group) at position 312 (p.Leu312Phe) (Figure 3A), located within the conserved UCR2 domain of PDE4D (Figure 3B).

It should be noted that another substitution at the same position (c.934C>G), resulting in a leucine-to-valine change (p.Leu312Val), was previously reported by Briet et al. (14). Similar to leucine, valine contains a non-polar aliphatic side chain. The authors demonstrated that this variant, along with three other variants, led to increased activation of PDE4D3 expressed in Chinese hamster ovary cells, thereby attenuating PTHR-cAMP-PKA signaling during development. The PDE4D isoform is highly expressed in the brain, particularly in the hippocampus. Mice lacking the *PDE4D* gene show enhanced long-term potentiation, which is associated with improved learning, memory, and even neural regeneration (16). This particular variant was identified in a 42-year-old woman who presented with severe intellectual disability, marked brachydactyly, brachymetatarsia, and brachymetacarpia, short stature (139 cm − 3.74 SDS), thickened bones, and obesity. Vitamin D supplementation in this patient normalized previously elevated parathyroid hormone (PTH) levels, that is, 107 pg./mL (normal range 10–65). Despite the phenotypic severity, the report did not include descriptions of facial phenotype or detailed radiographic findings of the knee joints.

Previous reports on ASD patients have demonstrated that the disorder incorporates acrodysostosis-like features such as more severe brachydactyly, brachymetatarsia, and brachymetacarpia with cone-shaped epiphyses, along with characteristic metaphyseal cup-shaped deformities of the knees. The phenotype consistently includes shortened and abnormally curved diaphyses of long bones, significant postnatal growth retardation (ranging from −3 to −8.8 SDS), delayed walking onset, and progressive flexion contractures of the knee joints. Notably, two reported ASD cases exhibited severe midface hypoplasia with nasal stenosis, resulting in clinically significant respiratory and swallowing difficulties, obstructive sleep apnea, and chronic otitis media (1, 6). In our reported case, the 7-year-old girl exhibited severe acrodysostosis with cone-shaped epiphyses of metacarpal bones and phalanges, combined with knee cup-shaped metaphyses containing embedded epiphyses, along with bowed left humerus and profound growth retardation (−4.81 SDS).

The general spectrum of the radiological changes found in the proband is typical for the condition and resembles the previously published data (1). Among the interesting radiological findings, a typical cupped metaphyseal deformity was not present in the early radiographic images. The previous publication did not present a series of radiographs demonstrating the natural history of radiological changes. The earliest published radiograph of the lower limbs at 7 months of age (6) demonstrates an obvious abnormality of the growth plates. In our patient, a radiograph obtained at 3 months of age revealed intact growth plates and a normal shape of the epiphyses. During the 3–7 year age range, cupped metaphyseal deformity progressed to typical scypho-deformity. Interestingly, radiographs of the hips were basically normal; the same pattern of radiologically normal hips can be noted in the previous publications (1–3, 6). Severe bowing of the left humerus and valgus deformity of the elbow secondary to the growth disturbances were found on the left side, demonstrating a mosaic pattern of damage. This unilateral humerus bowing was also noticed in the patient with ASD (6). In contrast, short metacarpals and phalanges were symmetrical and equally distributed among the fingers.

Our findings, demonstrating the postnatal origin of the scypho-deformity in ACD, are quite interesting and unique. It is unclear which factor initiates permanent damage to the growth plates, especially taking into account the mosaic (unilateral and/or asymmetrical) pattern. This unusual segmental distribution and atypical local pattern, characterized by predominantly central growth plate arrest leading to scypho-deformity, led to misdiagnosis with sequelae of neonatal sepsis and meningococcemia (6). Further studies can help to clarify the underlying mechanism of this selectivity.

Thinning of the isthmus of the corpus callosum and periventricular leukomalacia found on the brain MRI are within the spectrum of non-specific changes that can be found both in congenital and acquired CNS pathologies responsible for developmental delay and movement disorders (17).

The patient presented with a complete absence of speech, lack of social interaction, hyperexcitability, stereotypic hand-flapping movements, inability for self-care, and severely impaired wide-based gait with knee flexion and forward trunk inclination. Marked midface hypoplasia and nasal hypoplasia were present since birth, exacerbated by recurrent acute respiratory infections and febrile episodes. Notably, the patient showed no hormonal resistance. Literature review reveals that only two reported ASD cases demonstrated mild parathyroid hormone resistance, with one additional case showing peripheral hypothyroidism, while no other endocrine abnormalities have been documented (1, 2, 6).

To date, only six missense variants in the *PDE4D* gene have been identified in ultra-rare ASD cases, including our reported one (1, 2, 6, 7) (Table 1). Recently reported two ASD cases with *GNAS* variants further suggest genetic heterogeneity of this disorder (1). These findings indicate that ASD likely represents part of a broader spectrum of diseases characterized by overlapping clinical features and dysregulation of the intracellular cAMP-dependent signaling pathway. This disease continuum may encompass both forms of acrodysostosis (caused by *PRKAR1A* and *PDE4D* variants), Albright hereditary osteodystrophy, and pseudohypoparathyroidism (caused by *GNAS* variants).

**Table 1:** Phenotype and genotype of patient with acroscyphodysplasia with variants in *PDE4D* gene.

	Case 1 (1)	Case 2 (1)	Case 3 (1)	Case 4 (7)	Case 5 (6)	Case 6 (2)	Case 7 (2)	Our case 8
Sex, age	F, 3 y	F, 15 y	F, 9 m	F, 14 y	F, 14 y	N/A	N/A	F,7 y
Nationality	Philipino	Malaysian	Japanese	N/A	N/A	N/A	N/A	Chechen
		Chinese						
Variant	c.671C>T	c.671C>T	c.998 T>C	c.679G>T	c.913T>C	c.995T>C	c.997T>G	с.934C>T
	p.Thr224Ile	p.Thr224Ile	p.Ile333Thr	p.Ala227Ser	p.Ser305Pro	p.Phe332Ser	p.Ile333Met	p.Leu312Phe
Height (SDS)	NA	−8,8	NA	−3	NA	NA	NA	−4,81
Weight (SDS)	NA	−2	NA	NA	NA	NA	NA	+4,17
Obesity	NA	−	NA	NA	NA	NA	NA	+
Intellectual impairment	NA	+	+	NA	+	+	+	+
Phenotype	Hypoplastic nasal root, depressed nasal tip, short philtrum, down-turned mouth, iris heterochromia	Epicantus, nasal hypoplasia, depressed nasal bridge	Hypertelorism, nasal hypoplasia, depressed nasal bridge, mideface hypoplasia	Epicant, nasal and maxilla hypoplasia	Coarse bright red hair,brachycephalia, mideface hypoplasia, nasal hypoplasia, nasal stenosis, freckles	Nasal hypoplasia, flat face with malar hypoplasia, midface hypoplasia	Nasal hypoplasia, flat face with malar hypoplasia, midface hypoplasia	Hypertelorism, epicantus, nasal hypoplasia, depressed nasal bridge, mideface hypoplasia
Brachydactyly	+	+	+	+	+	+	+	+
Cone-shaped epiphyses	+	+	+	+	+	+	+	+
Radiological features	Tibial bowing, severe brachydactyly, severe cup-shaped large metaphyses of the knees	Unequal leg and arm lengths, severe brachydactyly, calvarial thickening, severe cup-shaped large metaphyses of the knees and shoulders	Severe brachydactyly, moderate cup-shaped large metaphyses of the knees and shoulders	Severe brachydactyly, moderate cup-shaped large metaphyses of the knees	Cone-shaped epiphyses of the right distal femur and proximal tibia, left distal femur contracture without evidence of schypho deformity, advanced bone age	Micromelia, severe brachydactyly with small phalanges and small metacarpals and metatarsals, cup-shaped metaphyses with embedded epiphyses on knees	Micromelia, severe brachydactyly with small phalanges and small metacarpals and metatarsals, cup-shaped metaphyses with embedded epiphyses on knees	Cone-shaped epiphyses with cup-shaped large metaphyses of the knees, humeral bowing
Endocrine changes	PTH resistance, normal thyroid function	Normal	Normal	Normal	Mild PTH resistance	NA	NA	Normal
Other	−	−	Hypotonia, apnea, failure to thrive	Enlargement of the toes	Gastrostomy tube for feeding in neonatal period, CPAP therapy	Non-specific MRI abnormalities	Non-specific MRI abnormalities	−

PTH, parathyroid hormone; TSH, thyroid stimulating hormone; NA, not available.

Our reported case significantly contributes to expanding both the phenotypic and genotypic spectrum of ASD, while providing critical evidence for its recognition as a distinct nosological entity within the classification of genetic skeletal disorders. The detailed clinical and molecular characterization of this patient enhances the current understanding of genotype–phenotype correlations and the molecular mechanisms underlying skeletal development, particularly those mediated by cAMP signaling. This case underscores the importance of comprehensive molecular analysis in atypical presentations of skeletal dysplasia and contributes to the evolving taxonomy of genetic disorders involving cAMP signaling defects. The accumulation of such carefully characterized cases will be essential for establishing a definitive diagnostic pathway and understanding the full clinical spectrum of ASD.

## Data Availability

The raw data supporting the conclusions of this article will be made available by the authors, without undue reservation.
